# First Report of *Trametes hirsuta,* Causal Agent White Rot in Avocado Trees Grown in the State of Michoacán, México

**DOI:** 10.3390/pathogens14060532

**Published:** 2025-05-26

**Authors:** Juan Mendoza-Churape, Ma. Blanca Nieves Lara-Chávez, Rosario Ramírez-Mendoza, César Ramiro Martínez-González, Hexon Angel Contreras-Cornejo, Yurixhi Atenea Raya-Montaño, Teresita del Carmen Ávila-Val, Margarita Vargas-Sandoval

**Affiliations:** 1 Laboratorio de Fitopatología, Facultad de Agrobiología “Presidente Juárez”, Universidad Michoacana de San Nicolás de Hidalgo, Uruapan 60170, Michoacán, Mexico; juan.churape@umich.mx (J.M.-C.); blanca.lara@umich.mx (M.B.N.L.-C.); yurixhi@umich.mx (Y.A.R.-M.); tere.avila@umich.mx (T.d.C.Á.-V.); 2 Colegio de Postgraduados, Km 36.5 Montecillo, Texcoco 56230, Estado de México, Mexico; rm_rosario@ciencias.unam.mx; 3 Tecnológico Nacional de México, Instituto Tecnológico de Ciudad Victoria, Ciudad Victoria 87010, Tamaulipas, Mexico; ramiro_mg.unam@ciencias.unam.mx; 4 Tecnológico Nacional de México, Instituto Tecnológico de Morelia, Morelia 58120, Michoacán, Mexico; marcenses@yahoo.com.mx; 5 Facultad de Biología, Universidad Michoacana de San Nicolás de Hidalgo, Morelia 58030, Michoacán, Mexico

**Keywords:** ITS, pathogenic fungus, *Persea americana*, polyporales, sporomas, wood decay fungus

## Abstract

México is the world’s leading producer of avocado, with 2,540,715 tons in the last year. *Trametes* spp. are macromycete fungi that rot wood. In 2022, in the state of Michoacán, México, sporomas of *Trametes* sp. were found in the trunks of avocado trees (*Persea americana* var. Hass) of 10 years old and older. The trees showed disease symptoms including yellowing of leaves, widespread defoliation, and wilting. It was observed that 10% of the infected trees were felled after heavy rains. In the place where the fungus settled, abundant cream-colored and cottony mycelium developed, causing “white rot”. The incidence of the disease in the sampled orchards was 60% in the tree population per hectare with 350 trees. The symptomatic trees studied were randomly selected from seven orchards. The collected fungal samples show typical structures corresponding to *Trametes* sp., including large sporomas, a pileus with a surface of concentric zones of various ocher tones, and a porous hymenium. The samples showed a 99% match with the species *Trametes hirsuta*. Laboratory bioassays of inoculation in fresh wood segments of avocado formed typical sporomas of the pathogen. Finally, the fungus was recovered and reisolated in vitro in PDA, and its identity was confirmed through the morphological characteristics and molecular tests. To the best of our knowledge, this article reports for the first time that *P. americana* cv. Hass and Mendez are new hosts for *T. hirsuta*. Therefore, the environmental and horticultural management conditions that favor the proliferation of *T. hirsuta* must be investigated.

## 1. Introduction

The genus *Trametes* (Polyporales: Polyporaceae) comprises wood-decay fungi, whose species are distributed in practically all terrestrial ecosystems colonized by trees that provide wood or food resources [1,2,3,4,5,6]. *Trametes* spp. are characterized by the presence of the sporoma firmly anchored to the trunk of the host tree, forming clusters. Although the occurrence of *Trametes* is more common in the autumn season, it can be seen throughout the year [7,8].

*Trametes* has also been described as a phytopathogenic fungus that possesses a complex system of wood-degrading enzymes. In living trees, this class of fungi can produce infections through wounds and subsequently colonize and degrade wood tissue, with an extraordinary ability to survive even after the tree’s death. In particular, *Trametes hirsuta* is one of the species that exhibit the aforementioned behavior [9,10]. For example, in 2017, *T. hirsuta* was reported for the first time as an agent of *Paulownia tomentosa* decline in Serbia [11]. Notably, when this fungus infects the tissue of its living host, it causes “white rot”, the name given to this disease that, after 3 to 5 years, commonly causes death.

Avocado is a fruit of high nutritional value that is consumed by cultural tradition in México, but large quantities of this food are also exported to other places in the world. In Michoacán, México, large tracts of land are being planted with avocado trees to supply the demanding market. However, the production of these fruits has been affected by several plant pathogens, including some emerging macromycetes [12,13,14,15].

Therefore, the objectives of this work were the following: (i) to formally report for the first time the infection caused by *T. hirsuta* in avocado trees, (ii) to describe this fungal species as an emerging pathogen in the “avocado belt” of the state of Michoacán, (iii) to establish an epidemiological record on the incidence of the infection in avocado orchards in this producing region, and (iv) to verify through laboratory bioassays that *T. hirsuta* is the causative agent of white rot in the woody tissue of avocado trees. To answer these questions, both phytopathogenic and molecular tests were used.

## 2. Materials and Methods

### 2.1. Study Sites

**Figure 1:**
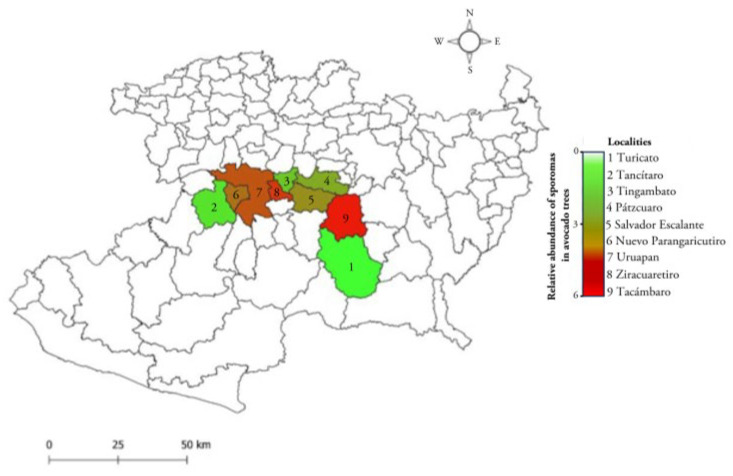
Sampling sites for *T. hirsuta* in avocado orchards in the state of Michoacán, México. In 2022, a population of 2450 trees was visited in the avocado belt, and 60% of the trees were observed to have formed sporomas. Detailed sporoma counts in trunks and branches were per-formed on N = 66 trees in these municipalities.

Fungal specimens were collected during the 2022 fall–winter seasons. From the sampling sites, sporomes attached to branches and trunks of avocado trees with symptoms of the disease (yellowing, defoliation, and dieback) were collected, as well as woody tissue with a white coloration due to the presence of the fungal mycelium. Each of the samples was placed in resealable Ziploc^®^ bags and labeled according to the order of collection, indicating the date and general data of the orchard. Sampling was carried out in avocado orchards in the area known as the “avocado belt” in Michoacán (19°13′ N, 101°55′ W), México (Figure 1). The sampled sites were localities with different agroclimatological conditions: Ziracuaretiro, Tacámbaro, Timgambato, Tancítaro, Pátzcuaro, Turicato, Salvador Escalante, Nuevo Parángaricutiro, and Uruapan. For an accurate record of the sampled sites, geographic data of longitude, latitude, and elevation were determined using a Global Positioning System (GPS-Garmin^®^).

### 2.2. Tree Selection and Sampling

The fungus was observed on 2450 adult avocado trees (*Persea americana* cv. Hass and Méndez) at the nine locations visited. The percentage of sporomas was calculated, considering the total inspected population as 100%. Fungal samples (n = 35 sporomes, 1 per tree, 5 per orchard) for morphophysiological, molecular, and phytopathological studies were randomly selected from 7 orchards. Tissue samples with white rot (n = 35, 1 per tree) were also collected from the same trees at the same sites.

The fungus was isolated on PDA (potato dextrose agar) culture medium, as well as on MEA (Malta Agar) for subsequent analysis.

For macroscopic characterization of the fungi, the synoptic keys described by Gilbertson and Ryvarden [1], Largent [16], and Kornerup et al. [17] were used. The fungal specimens and collected plant tissues were dried at 40–45 °C and stored in labeled cardboard boxes. The fungal specimens were deposited in the herbarium of the Phytopathology Laboratory of the Faculty of Agrobiology of the Michoacana University of San Nicolás de Hidalgo, Michoacán, México.

### 2.3. Characterization of the Fungus

To determine the microscopic characteristics of *Trametes*, longitudinal and transverse sections were made in the sporoma. The processed tissues were observed under a Nikon compound microscope. A calibrated micrometer was used to make measurements on the hyphal system, fibula, cystidia, basidia, and spores. Other characteristics such as shape, color, and wall thickness were also determined. Histochemical preparations were previously made on the fungal tissue to clarify it with KOH (5%). The size of the basidiospores was determined by measuring the length and width of 30 spores per sample, as described by Largent [16]. The range of length and width values is indicated with the symbol X. The variation in the length/width ratio of the basidiospores was determined and called Q.

For molecular identification of *Trametes*, genomic DNA was extracted using the CTAB method [17]. DNA was quantified with a Nanodrop 2000c (Thermo, Waltham, MA, USA). Dilutions of each sample at 20 ng were prepared to amplify the following five regions: (i) the nuclear ribosomal DNA (nrDNA) regions corresponding to the internal transcribed spacers (ITSs) 5.8S rDNA-ITS1 and rDNA-ITS2 (primers ITS5-ITS4), (ii) the section of DNA encoding the large ribosomal subunit 28S rRNA (primers LROR-LR3), both molecular markers as described by White et al. [18], (iii) the largest subunit of partial RNA polymerase II (rpb1), (iv) the second largest subunit of RNA polymerase II (rpb2; primers RPB2-5F/RPB2-7cR), and (v) translation elongation factor 1-α (tef1; primers 983F-2218R), all three protein-coding genes as described by Sung et al. [19].

The PCR reaction mix was prepared in a final volume of 13 μL containing 1x Taq DNA polymerase enzyme buffer, 0.8 mM of deoxynucleoside triphosphate (0.2 mM each), 100 ng of DNA, 20 pmol of each primer, and 2 units of GoTaq DNA (Promega, Madison, WI, USA). The PCR amplification conditions for ITSs and LSUs were as follows: 3 min at 94 °C, followed by 35 cycles of 95 °C for 30 s, 55 °C for 1 min, and 72 °C for 1 min, with a final extension at 72 °C for 10 min. The amplification condition for tef1 consisted of an initial denaturation for 5.30 min at 95 °C, followed by 35 cycles of 94 °C for 1 min, 57 °C for 30 s, and 72 °C for 1.30 min, with a final extension at 72 °C for 10 min; for rpb1 and rpb2, it consisted of an initial denaturation for 3 min at 94 °C, followed by 35 cycles of 95 °C for 1 min, 52 °C for 2 min, and 72 °C for 1 min, with a final extension at 72 °C for 10 min [19].

All PCR reactions were carried out in a PTC-200 Peltier thermal cycler (BIORAD, Mexico City Mexico). PCR products were verified by agarose gel electrophoresis. Gels were run for 1 h at 95 V cm^−3^ in 1.5% agarose and 1× TAE buffer (Tris Acetate-EDTA). The gel was stained with GelRed (Biotium, Fremont, CA, USA) and bands were visualized on an Infinity 3000 transilluminator (Vilber Lourmat, Eberhardzell, Germany). Amplified products were purified with the ExoSAP Purification kit (Affymetrix, Santa Clara, CA, USA), following the manufacturer’s instructions. They were quantified and prepared for the sequencing reaction using a BigDye Terminator v.3.1 (Applied Biosystem, Foster City, CA, USA). These products were sequenced in both directions using an Applied Biosystem model 3730XL (Applied BioSystems, Foster City, CA, USA). To generate consensus sequences, the sequences of both strands of each gene were analyzed, edited, and assembled using BioEdit version 7.0.5 [20]. These consensus sequences were compared with those deposited in GenBank at the National Center for Biotechnology Information (NCBI), using the BLASTN 2.2.19 tool [21]. The sequences were subjected to standard Nucleotide BLAST searches in GenBank to determine the primary identity of the fungal isolates. *Lopharia cinerascens* [4] was used as an outgroup.

To study phylogenetic relationships, our newly produced sequences from nine *Trametes* individuals were added to the reference ITS, LSU, rpb1, rpb2, and tef1 sequences deposited in the NCBI database (http://www.ncbi.nlm.nih.gov/genbank/ accessed 24 January 2024). Each region was independently aligned using the online version of MAFFT v7 [22,23,24]. The alignments were reviewed in PhyDE v. 10.0 [25], followed by minor manual adjustments to ensure character homology between taxa. Matrices were formed for the ITS region with 99 taxa (685 characters), for the LSU region with 64 taxa (585 characters), for rpb1 with 36 taxa (883 characters), for rpb2 with 42 taxa (775 characters), and for tef1 with 23 taxa (599 characters). Eleven partitioning schemes were established: one for the ITS region, one for the LSU region, three to represent the three codon positions of the rpb1 gene region, three to represent the three codon positions of the rpb2 gene region, and three to represent the three codon positions of the tef1 gene region, which were established using the option to minimize stop codons with Mesquite v3.2 [26,27].

### 2.4. Data Analysis

The data were analyzed using maximum parsimony, maximum likelihood and Bayesian inference. Maximum parsimony analyzes were carried out in PAUP* 4.0b10 [28] using the heuristic search mode, 1000 random starting replicates, and TBR branch swapping, with MULTREES and Collapse on. Bootstrap values were estimated using 1000 bootstrap replicates under the heuristic search mode, each with 100 random starting replicates. Maximum likelihood analyses were carried out in RAxML v. 8.2.10 [29] with a GTR + G model of nucleotide substitution.

To assess branch support, 10,000 rapid bootstrap replicates were run with the GTRGAMMA model. Bayesian inference was carried out in MrBayes v. 3.2.6 ×64 [30] with four chains, and the best model for alignment was sought using PartitionFinder [31,32]. Phylogenetic analyses were performed using MrBayes v3.2.6 ×64 [28]. The information block for the matrix included two simultaneous runs of Monte Carlo chains, temperature set to 0.2, and 10 million sampling generations (standard deviation ≤0.1). Chain convergence was visualized in Tracer v.1.6 [32]. The remaining trees were used to calculate a 50% majority-rule consensus topology and posterior probabilities (PPs). Trees were visualized and optimized in FigTree v. 1.1.4 [33] and edited in Adobe Illustrator (Adobe Systems, Inc., San Jose, CA, USA).

### 2.5. Bioassays for Pathogenicity

Pathogenicity tests were performed by inoculating fungal mycelium (0.5 g of a pure *T. hirsuta* colony grown for 15 days on solidified nutrient medium (MEA)) on six-month-old avocado seedlings (n = 63 for three fungal isolates, 21 plants for each one) were inoculated at the root/stem intersection with 0.5 g of *T. hirsuta* mycelium. Seedlings without fungal inoculation were used as controls (n = 9). Fungal inoculation experiments were also performed on living woody tissue (13 segments of 15 cm and Ø = 10 cm per treatment, n = 13 per isolate [N = 9 control fragments]), freshly cut from 10-year-old trees grown under field conditions. It was placed in the central part of the section of the wood fragment held by a previously sterilized thumbtack. Likewise, inoculation was performed on wood fragments with MEA nutrient medium disks but without fungal mycelium for the controls. They were left in laboratory conditions at an average temperature of 18.5° C for 8 weeks. We used a randomized experimental design to evaluate the severity of disease symptoms, visually evaluating fungal growth and the texture, color, signs, and progression of disease in fresh wood.

## 3. Results

**Figure 2:**
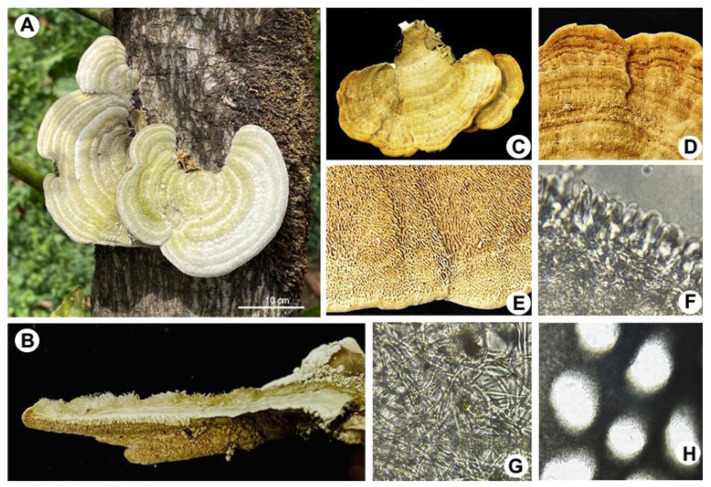
Macroscopic morphology of *Trametes* sp. (**A**) Presence of *Trametes* sp. in the form of a sporoma attached to the trunk of a 12-year-old avocado tree grown in an orchard in Michoacán, México. (**B**,**C**) Sporoma in its cross section and adaxial plane, respectively. (**D**) Pileus with surface of concentric zones of various ocher tones and covering of bristly villi. (**E**) Hymenium with irregular pores measuring 3 or 4 per mm, whitish to cream in color, with grayish hues and areas with ochraceous tones. (**F**) Basidia. (**G**) Thin-walled, hyaline hyphae (2.5–7.5 cm). (**H**) Round and angular pores.

Table 1 records the geographical localization of the orchards, their altitude, and the annual precipitation data, indicating that infected trees are ubicated in the avocado belt of Michoacán. Interestingly, sporomas of *Trametes* sp. were found attached in the trunks of avocado trees of 10 years old and older (Figure 2). The incidence of the disease in the sampled orchards was 60% per hectare with 350 trees. It was observed that less than 10% of the infected trees were felled after seasonal heavy rains. In the place of the tree where the fungus settled, abundant cream-colored and cottony mycelium formed, causing white rot. Each tree was found to have 2.89 ± 0.19 sporomas; 0.81 ± 0.07 on the trunk and 2.07 ± 0.14 on the branches, respectively (Figure 2). However, strictly evaluating the abundance of sporomas per tree, the localities with the highest counts were Tacámbaro and Ziracuaretiro, with five and six sporomas, respectively (Figure 1).

**Figure 3:**
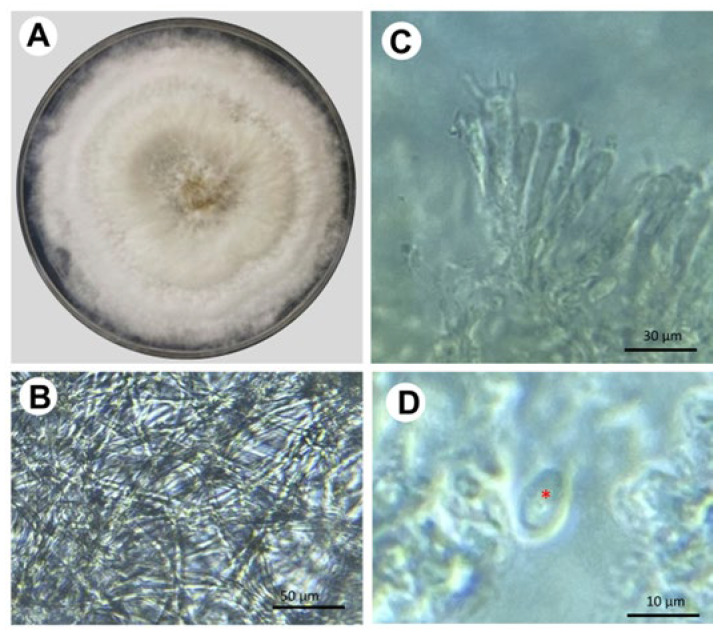
Mycelial and microscopic morphology of *Trametes* sp. (**A**) Fungal colony formed by the mycelial form of *Trametes* in PDA. (**B**) Hyaline mycelium from a 15-day-old fungal colony. (**C**) Basids (15–20 × 5–7 µm) and (**D**) Spore (8 × 5 µm); the red asterisk shows the localization of such structure. Sample collection in the avocado orchards took place during the rainy season of 2022.

The fungal samples show typical structures corresponding to *Trametes* sp., including large sporomas, a pileus structure with a surface of concentric regions of different ocher tones, and a porous hymenium (Figure 2). In vitro isolation of the fungus was carried out from the sporoma and tissues of infected trees. The fungal strains were maintained on potato dextrose agar (PDA) at 28 °C, where they formed a well-defined and confluent colony. After 20 days of incubation, cottony mycelium was formed, and it developed a slightly yellowish, opaque circular colony (Figure 3). Microscopic analysis of the fungus showed cream-colored cylindrical spores measuring an average of 8 × 5 μm (270 structures counted per isolate [N = 9]; Figure 3).

**Figure 4:**
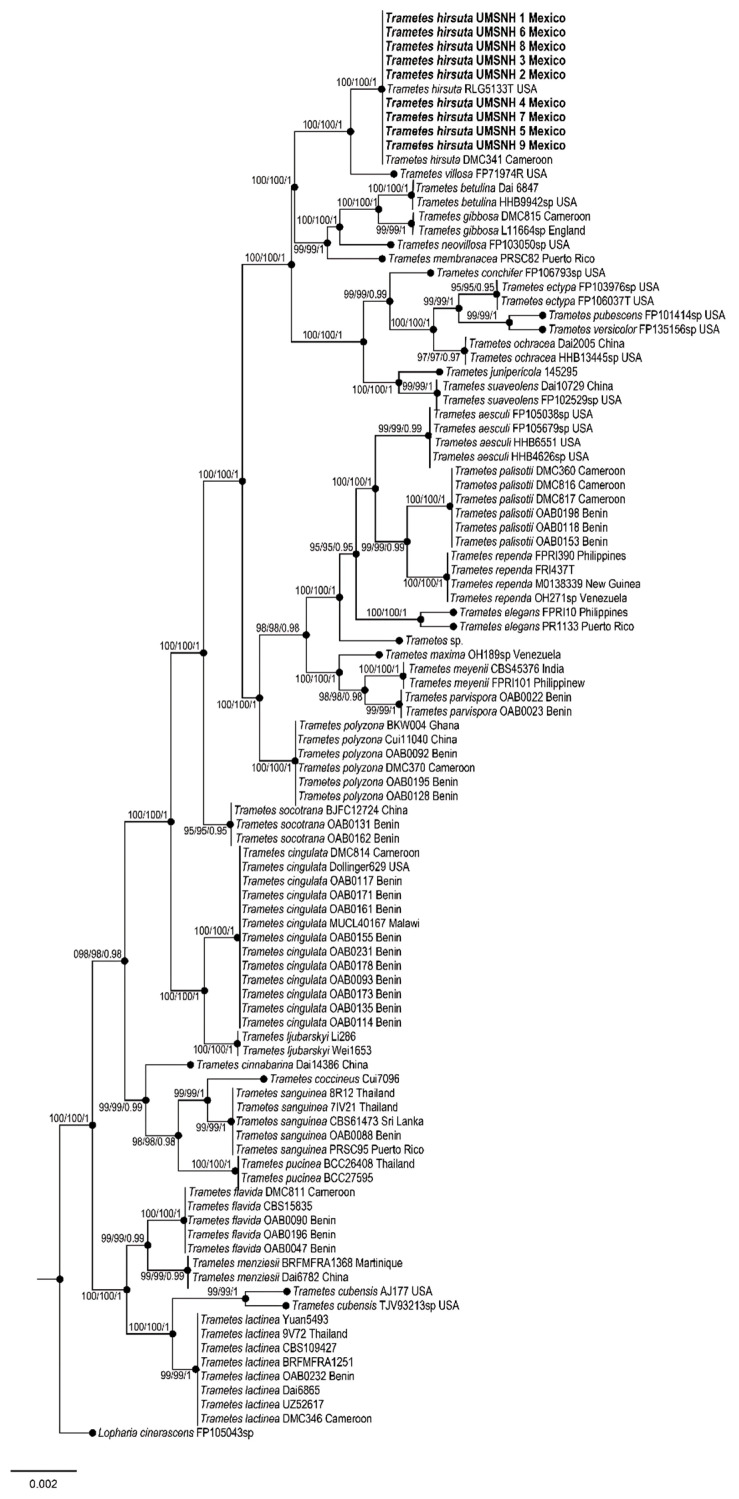
Phylogeny for *T. hirsuta* based on the concatenated ITS, LSU, rpb1, rpb2, and tef1 sequences. No significant conflict (bootstrap value > 80%) was found between the topologies obtained through separate phylogenetic analyses. The scale bar indicates the expected number of nucleotide substitutions per site.

For molecular characterization of the phytopathogen, PCR reactions were performed, and a phylogenetic tree was constructed (Figure 4; Table 2). In the Bayesian analysis, the standard deviation between chains stabilized at 0.001 after 3.5 million generations. No significant changes were observed in the tree topology trace or in the accumulated division frequencies of the selected nodes after approximately 0.25 million generations, which were discarded as 25% burn-in. Genetic analyses showed a 99% match with the species *T. hirsuta* (Figure 4).

**Figure 5:**
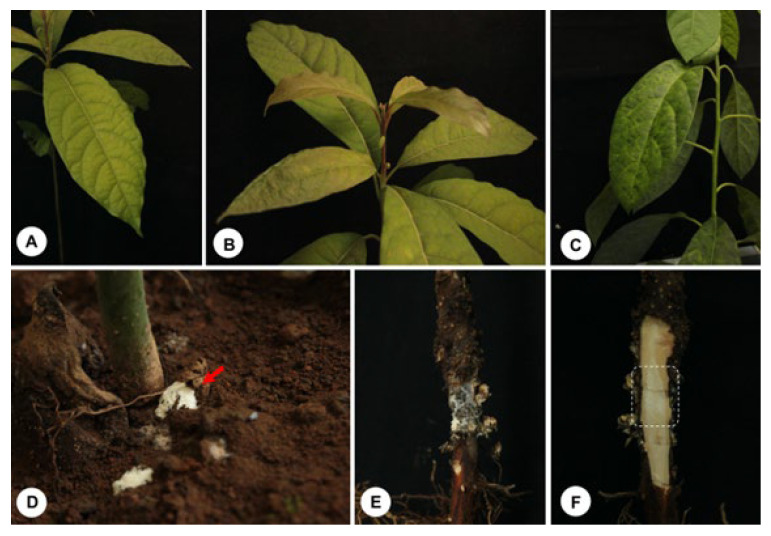
Pathogenicity tests of *T. hirsuta* on avocado seedlings. (**A**) Foliage of the control treatment. Signs of the disease caused by the fungus: (**B**) chlorosis and (**C**) wilting. (**D**) Establishment of the fungus and formation of a dense body of fungal biomass associated with the host stem, red arrow phytopathogen mycelium. (**E**) Invasion and evident necrosis of the bark and part of the cabium of the young tissue of avocado seedlings. (**F**) Longitudinal section of the stem infected with *T. hirsuta* showing the softening of the woody tissue caused by the white rot (white dotted) of *T. hirsute*.

To fulfill Koch’s postulates, 6-month-old avocado seedlings were inoculated with *T. hirsuta*. After 6 weeks, the seedlings showed signs of disease, such as chlorosis and generalized wilting of the leaves. When the root system was examined, the fungus was found to cause root rot (Figure 5). Interestingly, a voluptuous body of fungal biomass was observed in the lowest part of the stem of the seedlings, suggesting the formation of the sporoma primordium. A longitudinal section of the woody tissue of the tomato seedlings revealed softening and white rot in the tissue where the *T. hirsuta* mycelium had colonized (Figure 5).

**Figure 6:**
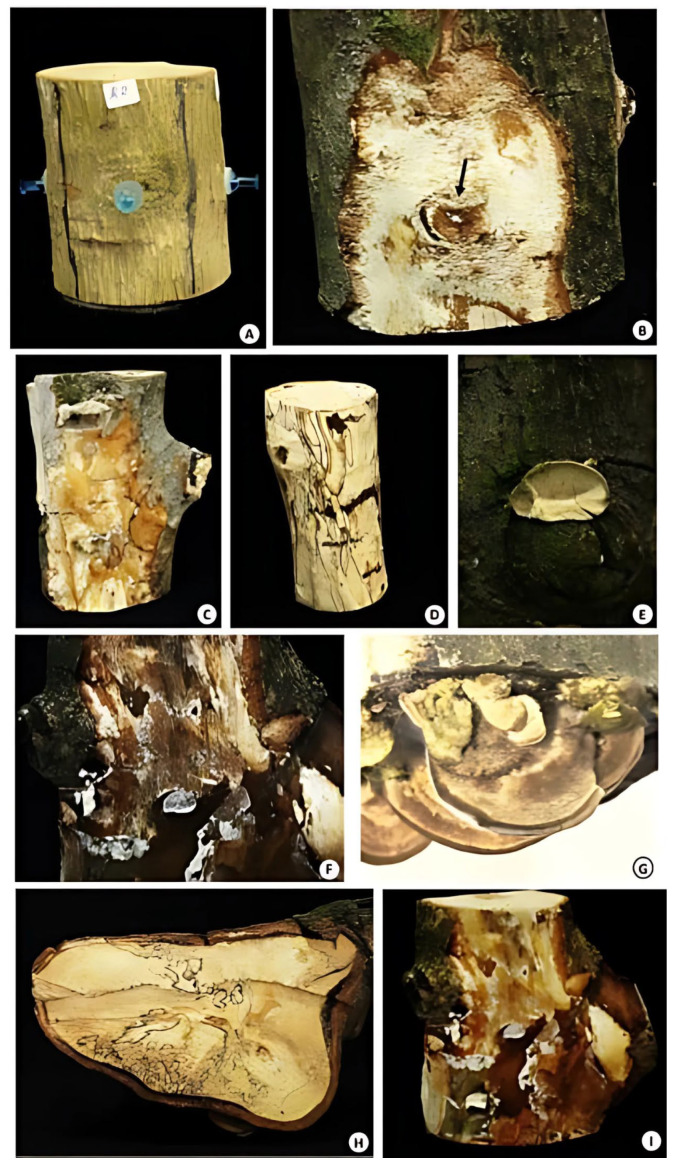
Pathogenicity tests for *T. hirsuta* in avocado woody tissue. (**A**) Control avocado trunk (control). (**B**) Cross section with lesion of the infection site where the phytopathogen was inoculated (black arow). (**C**,**F**,**I**) Spongy-looking white rot with invasion of mycelium in the sapwood of the wood. (**D**) External diagnosis of the wood infection: pale coloration of the tissue is a characteristic sign of the white rot. (**E**) Primordium of *T. hirsuta*. (**G**) Sporomas obtained from inoculation, an evident sign of the disease. (**H**) Localized necrosis and white rot in the sapwood, heartwood, and pith of the tissue. The experiment was conducted twice with simulated results.

To confirm whether isolates also exhibited wild-type infection behavior in the field, segments of fresh wood from avocado trees grown under field conditions were inoculated with *T. hirsuta* mycelium, and non-inoculated wood segments were used as controls. The first symptoms appeared 21 days after inoculation, which include the formation and development of mycelium on the woody tissue, causing white rot, with an evident change in the structure and texture of the tissue at 48 days and the appearance of fruiting bodies. It was clearly evident after 60 days that 98% of the inoculated samples developed clear signs and symptoms of the infection, and 77% of the cases developed severe mycosis, completely rotting the wood, as shown in the pathogenicity tests. After eight weeks, the formation of sporomas with dimensions of 2 × 3 × 0.2 cm on average was observed, where 100% of the inoculated sites presented white rot at the inoculated site (Figure 6). Finally, the pathogen was recovered and reisolated in vitro on PDA, and its identity was confirmed through morphological characteristics and molecular tests.

## 4. Discussion

Due to the number of trees showing signs and symptoms of disease in the “avocado belt” in Michoacán, México, a new phytosanitary alert emerged among producers. Phytopathological studies showed that the etiological agent of disease is *Trametes*, a fungus that forms sporomas on the trunks of trees ~10 years old and older. Several species of *Trametes* are part of the mycoflora of the state of Michoacán [34], which can be recognized in the field by their gray and brown sporomas [10]. Before 2022, these species did not cause phytosanitary problems in the region.

In the nine sampled locations, the highest number of sporomas was detected on the branches of trees infected with *Trametes*, revealing that the fungus has an efficient dispersal mechanism in the environment, mediated in part by air currents. Interestingly, comparing the average number of sporomas in the trees of the different locations, it was observed that precipitation is an important environmental factor that could favor the spread of fungus. Turicato, a locality with fewer sporomas per tree and low rainfall, was recorded, while the number of sporomas and rainfall were higher in the other locations, as in Ziracuaretiro. Interestingly, an examination of the map we constructed in this article shows that the nine localities where *Trametes* was found are interconnected, which strongly suggests the spread of the pathogen in the avocado belt of the state of Michoacán. However, it is necessary to investigate the environmental and horticultural management conditions that favor the proliferation of *Trametes* in more depth.

Molecular analysis confirmed that the species associated with avocado tree tissues was *T. hirsuta*. Several reports suggest that *T. hirsuta* is closely related to *Trametes villosa* [4,35], which is also supported by this work. In a strict census of the Polyporaceae family in México, it was shown that *T. hirsuta* and *T. villosa* are among the most widely distributed species in the country [36]. The latter species has the great ability of simultaneously depolymerizing lignin (a structural support component and protective barrier against pathogens), cellulose, hemicellulose, and pectin [37].

Interestingly, white rot lesions were clearly observed where this fungus colonizes, either in seedling tissue or in fresh wood segments. Previously, *T. hirsuta* was known as a saprobic fungus with a specialized enzyme complex for degrading lignin and other essential wood compounds [38,39]. During mycosis, white rot is due to the action of lignolytic enzymes that decompose numerous aromatic and structural components of wood [40]. Laccases (p-diphenol: dioxygen oxidoreductases) are a class of enzymes produced by most white rot fungi, where these enzymes degrade lignin, a complex polymer present in the cell walls of wood and bark, which is made up of phenylpropanoid units of three classes including coniferyl, sinapyl, and p-coumaryl alcohols [41,42]. Mn-oxidizing peroxidases also play an important role in lignin degradation [43].

*T. hirsuta* degrades 39.8% of lignin after 11 days, when this heteropolymer is the sole carbon source for the fungus; lignin degradation by this species includes the cleavage of Cα-Cα bonds and interunit β-β bonds [44]. Lignin degradation allows carbon to be reincorporated into the soil and activates the biogeochemical cycle of this element [45]. In addition to ligninolytic enzymes, *T. hirsuta* also produces cellobioses, cellulolytic enzymes, glyoxal oxidase (an H_2_O_2_-generating enzyme), hemicellulolytic enzymes, and xylanases [46], which are part of the molecular machinery necessary to degrade the structural components of wood.

On a physiological level, wood decay by *T. hirsuta* is characterized by cellular disaggregation, the formation of circular cavities, and pit erosion, indicating delignification [47]. This same fungus can form erosion channels through cellulose microfibrils, a characteristic of soft rot decay [47]. Our experiments revealed that *T. hirsuta* produced signs and symptoms in living wood similar to those observed in the field, highlighting the development of abundant cottony white mycelium that completely covers the affected areas between the heartwood and sapwood of the plant tissue. Notably, after 60 days, the formation of basidiomes attached to wood fragments was also observed, demonstrating that *T. hirsuta* is indeed the cause of white rot in avocado trees. *Trametes* is a xylotrophic fungus capable of surviving on fallen branches or wood debris [48].

Under field conditions, in seven avocado orchards, we visited at least 2450 trees, of which 1470 had anchored sporomas of *T. hirsuta*, representing 60% of the observed population. This fact demonstrates that the fungus caused a plant epidemic in the region. This negative event is partly due to poor forestry and agricultural practices, as occurs in other forest outbreaks [49], and to the change in land use for large-scale avocado cultivation [50], which consequently alters the habitat and natural behavior of *Trametes* as a forest necromass wood-destroying fungus in the pine–oak forests of the region and provokes a change in the lifestyle of *T. hirsuta* as an emerging phytopathogen. This fact is very likely because in biochemical terms, white rot fungi have the ability to modulate their metabolism by controlling the tricarboxylic acid/glyoxylate bicycle [45], which can occur during the growth of the fungus on its substrate.

It was recently reported that changes in the substrate composition of *T. hirsuta*, following the addition of sawdust, significantly modified the production of volatile terpenes [51]. Furthermore, the type of substrate used as a carbon source and temperature are two key factors that considerably modulate gene expression in *Trametes pubescens*; it is noteworthy that temperature alters carbon metabolic enzymes and glycoside hydrolases in this species [52]. It is not common to observe *T. hirsuta* behaving as an endophytic fungus inducing beneficial effects on its host, but depending on the environmental conditions, the fungus modifies its behavior. For example, a strain of *T. hirsuta* was isolated from the roots of Chenopodium album and promoted the growth of *Triticum aestivium*, even in the presence of Pb [53]. Therefore, the change in the behavior of *T. hirsuta,* as a pathogenic fungus beneficial to plants or as a decomposer of organic matter, is an outstanding characteristic of this organism depending on the environmental conditions prevailing in its ecological niche.

An important study showed that *Trametes* sp., *Trametes versicolor,* and *T. villosa* are part of the wood-decomposing basidiomycete species of the Andean Forest in Boyacá, Colombia, consisting of oaks [54], which indicates that forest ecosystems, common in México, are the ecological niche of fungal species with high lignocellulolytic activity [4,45]. Currently, *T. hirsuta* is present in America, Asia, and Europe [4,8,55,56], although the site of origin of this species and whether all these isolates belong to the same lineage, as well as its dispersal mechanism, are unknown.

In part, the phytosanitary problem arises because the fungus is present in its natural habitat, and when avocado trees are introduced as a new species into the soil, the fungus continues its ecological function, finding its new host in the trees. The problem of fungal infection is further complicated because the new avocado trees grown in these sites are now susceptible to extreme climatic conditions, such as low temperatures and drought. The latter factor is caused by climate change and the excessive use of water for the maintenance and formation of new avocado orchards, which causes severe water shortages throughout the producing region and increases the susceptibility of the entire tree population to damage caused by *T. hirsuta*, as occurs with other pathogens in other plants [49].

Another aspect to consider is the fact that during and after weed pruning in avocado orchards, the tree’s integrity is neglected, and physical damage to some part of the tree is reported, with wounds being an entry route for *Trametes*. In our previous sampling activities in avocado orchards (15 years ago), we observed that *Trametes* sp. colonized tissue damaged or weakened by external factors, such as breaks in the sapwood of the woody tissue associated with drastic temperature fluctuations or mechanical damage from pruning, which have been direct entry points for the phytopathogen (personal communication). Furthermore, once pruning is performed, whether for sanitation or rejuvenation of trees, the first symptoms and the appearance of fruiting bodies are recorded between two to three years after the penetration and development of the infection.

The first case of *T. hirsuta* causing deterioration of a *Paulownia tomentosa* plantation in Serbia also emerged from monitoring the health of trees in the area of the Medveja Village, near the town of Trstenik. This plantation was 5 years old and was constructed with one-year-old seedlings, where the fungus also caused white rot [11]. Bioassays we performed on fresh wood segments showed that the fungus does not produce fruiting bodies immediately after primary infection, and signs of infection appear when a large proportion of the wood is already colonized by the plant pathogen’s mycelium.

Liers et al. [57] indicated that the specialization of white rot fungi such as *T. hirsuta* has been originally described with a preference for hardwoods, but without affinity for any particular plant species, which explains the infection of the fungus in avocado orchards, since the wood of this tree is semi-hard and not very resistant, which facilitates its colonization and degradation by *Trametes*. A critical analysis of the literature revealed that in the central Black Earth region of Russia, *T. hirsuta* is a fungus that grows on fruit tree species [9], which also constitutes a background on the behavior of this organism affecting the flora of agricultural interest in temperate ecosystems such as the pine–oak forests of Michoacán.

The environmental and horticultural management conditions that favor the proliferation of *T. hirsuta* should be investigated and phytosanitary protocols should be established to prevent its spread to other avocado-producing regions. This fact is very important because practically all terrestrial ecosystems with tree species have the environmental conditions to allow the reproduction and proliferation of these class of fungi, according to the map of *Trametes* diversity worldwide presented by Olou and co-workers [4]. A promising alternative for the control of *T. hirsuta* is the use of biological control agents such as *Trichoderma*, which under experimental conditions managed to significantly inhibit the growth of the pathogen [58].

Finally, to our knowledge, this work reports for the first time that *P. americana* is a new host for *T. hirsuta* and that this fungal species is a new pathogen of avocado trees grown in Michoacán, the main producing state in México and worldwide. Furthermore, this manuscript establishes the epidemiological basis for the pathogen’s proliferation in the region and sets a precedent for monitoring and preventing the spread of *T. hirsuta* to other regions in Michoacán and worldwide where avocado seedlings are exported from nurseries. In the first reports of the disease, it was associated with severe death, and it is suggested that once the disease has been identified, according to the signs and symptoms already established in this work, cultural work such as pruning should be carried out, where not only the inoculum is eliminated but also an environment not conducive to the development of the pathogen is maintained. In initial cases of infection, after the removal of damaged tissue, applications of antifungal chemical molecules belonging to the Triazoles group should be made, due to their systemic effect and their biological effectiveness in inhibiting the growth and development of a significant number of phytopathogenic fungi [59].

## Figures and Tables

**Table 1:** Locations of avocado orchards in Michoacán, México, where *Trametes* sp. sporomas were collected are presented in bold font.

Municipality	Altitude	Precipitation	Coordinates	Incidence of Diseases by Location	Strain/Isolates
Ziracuaretiro	1571	1200	19.48°70′34″ N; −101.96°31′72″ W	1.71%	UMSNH 6
	1571	1200	19.46°16′25″ N; −101.94°46′33″ W	1.71%	UMSNH8
Tacámbaro	1654	1172	19.36°63′89″ N; −101.44°91′97″ W	3.42%	UMSNH1
Timgambato	1982	1100	19.50°11′47″ N; −101.89°14′25″ W	1.14%	UMSNH5
Tancítaro	2079	1800	19.33°87′34″ N; −102.34°54′09″ W	1.4%	UMSNH9
Pátzcuaro	2329	1200	19.46°80′28″ N; −101.69°85′30″ W	2.28%	UMSNH3
Turicato	1891	849	19.14°07′43″ N; −101.59°46′08″ W	1.71%	UMSNH2
Salvador Escalante	2200	1600	19.42°01′74″ N; −101.76°63′53″ W	2.57%	UMSNH8
Nuevo Parangaricutiro	1965	1800	19.43°67′40″ N; −102.18°15′93″ W	2.28%	UMSNH7
Uruapan	1620	1600	19.44°30′28″ N; −102.02°15′48″ W	4.57%	UMSNH4

Altitude is expressed in meters (m), The values of precipitation are the annual means represented in millimeters/metre^2^.

**Table 2:** GenBank accessions numbers of the sequences employed in the phylogenetic analyses. Sequences data created in this study are presented in bold font.

			GenBank Accesions				
Species Name	Isolate	Origin	ITS	LSU	*rpb1*	*rpb2*	*tef1*
*Trametes aesculi*	HHB4626sp	USA	JN164950	-----	KF573173	KF573134	KF573083
*Trametes aesculi*	FP105679sp	USA	JN164944	JN164799	JN164833	JN164861	JN164899
*Trametes aesculi*	HHB6551sp	USA	JN164938	-----	KF573172	KF573163	KF573082
*Trametes aesculi*	FP105038sp	USA	JN164951	-----	KF573174	KF573135	KF573081
*Trametes betulina*	HHB9942sp	USA	JN164983	JN164794	-----	JN164860	-----
*Trametes betulina*	Dai6847		KC848305	KC848390	-----	-----	-----
*Trametes cingulata*	MUCL: 40167	Malawi	JN645075	-----	-----	-----	-----
*Trametes cingulata*	Dollinger 629	USA	KY264043	-----	-----	-----	-----
*Trametes cingulata*	DMC814	Cameroon	KC589133	KC589159	-----	-----	-----
*Trametes cingulata*	OAB0135	Benin	MK736973	-----	-----	-----	-----
*Trametes cingulata*	OAB0117	Benin	MK736972	-----	-----	-----	-----
*Trametes cingulata*	OAB0093	Benin	MK736970	-----	-----	-----	-----
*Trametes cingulata*	OAB0114	Benin	MK736971	MK736950	-----	-----	-----
*Trametes cingulata*	OAB0161	Benin	MK736975	MK736951	-----	-----	-----
*Trametes cingulata*	OAB0155	Benin	MK736974	-----	-----	-----	-----
*Trametes cingulata*	OAB0171	Benin	MK736976	MK736952	-----	-----	-----
*Trametes cingulata*	OAB0173	Benin	MK736977	MK736953	-----	-----	-----
*Trametes cingulata*	OAB0178	Benin	MK736978	MK736954	-----	-----	-----
*Trametes cingulata*	OAB0231	Benin	MK736979	MK736955	-----	-----	-----
*Trametes cinnabarina*	Dai 14386	China	KX880629	KX880667	KX880818	KX880854	-----
*Trametes coccinea*	Cui 7096		KC848330	KC848414	-----	-----	-----
*Trametes conchifer*	FP106793sp	USA	JN164924	JN164797	JN164823	JN164849	-----
*Trametes cubensis*	TJV93-213sp	USA	JN164923	JN164798	JN164834	JN164865	-----
*Trametes cubensis*	AJ177	USA	JN164905	-----	-----	-----	-----
*Trametes cubensis*	UZ526-17	Malasya	MF363158	-----	-----	-----	-----
*Trametes ectypa*	FP10397sp	USA	JN164961	-----	-----	-----	-----
*Trametes ectypa*	FP106037T	USA	JN164929	JN164803	JN164824	JN164848	-----
*Trametes elegans*	PR1133	Puerto Rico	JN164937	-----	KF573178	KF573139	KF573075
*Trametes elegans*	FPRI10	Philippines	JN164973	-----	-----	KF573138	KF573074
*Trametes elegans*	FP150762	Belize	JN164928	-----	-----	KF573137	KF573076
*Trametes flavida*	OAB0047	Benin	MK736966	MK736946	-----	-----	-----
*Trametes flavida*	OAB0090	Benin	MK736967	-----	-----	-----	-----
*Trametes flavida*	OAB0196	Benin	MK736968	MK736947	-----	-----	-----
*Trametes flavida*	DMC811	Cameroon	KC589130	KC589156	-----	-----	-----
*Trametes flavida*	CBS 15835		MH855616	MH867126	-----	-----	-----
*Trametes gibbosa*	DMC815	Cameroon	KC589144	KC589164	-----	-----	-----
*Trametes gibbosa*	L11664sp	England	JN164943	JN164800	JN164831	JN164859	-----
*Trametes hirsuta*	DMC341	Cameroon	KC589146	KC589166	-----	-----	-----
*Trametes hirsuta*	RLG5133T	USA	JN164941	JN164801	JN164829	JN164854	-----
***Trametes hirsuta***	**UMSNH 1**	**Mexico**	**OR492495**	**OR492546**	**PQ961250**	**PQ972541**	**PQ978426**
***Trametes hirsuta***	**UMSNH 2**	**Mexico**	**OR492496**	**OR492547**	**PQ961251**	**PQ972542**	**PQ978427**
***Trametes hirsuta***	**UMSNH 3**	**Mexico**	**OR492497**	**OR492548**	**PQ961252**	**PQ972543**	**PQ978428**
***Trametes hirsuta***	**UMSNH 4**	**Mexico**	**OR492498**	**OR492549**	**PQ961253**	**PQ972544**	**PQ978429**
***Trametes hirsuta***	**UMSNH 5**	**Mexico**	**OR492499**	**OR492550**	**PQ961254**	**PQ972545**	**PQ978430**
***Trametes hirsuta***	**UMSNH 6**	**Mexico**	**OR492500**	**OR492550**	**PQ961255**	**PQ972546**	**PQ978431**
***Trametes hirsuta***	**UMSNH 7**	**Mexico**	**OR492501**	**OR492552**	**PQ961256**	**PQ972547**	**PQ978432**
***Trametes hirsuta***	**UMSNH 8**	**Mexico**	**OR492502**	**OR492553**	**PQ961257**	**PQ972548**	**PQ978433**
***Trametes hirsuta***	**UMSNH 9**	**Mexico**	**OR492503**	**OR492554**	**PQ961258**	**PQ972549**	**PQ978434**
*Trametes junipericola*	145295 (O)		KC017758	KC017763	-----	-----	-----
*Trametes lactinea*	DMC346	Cameroon	KC589126	KC589152	-----	-----	-----
*Trametes lactinea*	CBS 109427	Taiwan	MH862825	-----	-----	-----	-----
*Trametes lactinea*	LIP: GUY09	French Guiana	JN645069	-----	-----	-----	-----
*Trametes lactinea*	Dai6865		KC848327	KC848411	-----	-----	-----
*Trametes lactinea*	OAB0232	Benin	MK736983	MK736948	-----	-----	-----
*Trametes lactinea*	BCC 33266	Thailand	GQ982888	GQ982881	-----	-----	-----
*Trametes lactinea*	Yuan 5493		KC848320	KC848404	-----	-----	-----
*Trametes ljubarskyi*	Wei1653		KC848332	KC848416	-----	-----	-----
*Trametes ljubarskyi*	Li286		KC848331	KC848415	-----	-----	-----
*Trametes maxima*	OH189sp	Venezuela	JN164957	JN164804	JN164816	JN164864	-----
*Trametes membranacea*	PRSC82	Puerto Rico	JN164945	JN164805	JN164832	JN164857	-----
*Trametes menziesii*	BRFM FRA	Martinique	JN645103	-----	-----	-----	-----
*Trametes menziesii*	Dai6782		KC848289	KC848374	-----	-----	-----
*Trametes meyenii*		Philippines	JN164933	-----	KF573179	KF573145	-----
*Trametes meyenii*	CBS45376	India	MH860991	MH872762	-----	-----	-----
*Trametes neovillosa*	FP103050sp	USA	JN164958	JN164806	JN164835	JN164862	-----
*Trametes ochracea*	HHB1344sp	USA	JN164954	JN164812	JN164826	JN164852	-----
*Trametes ochracea*	Dai2005	China	KC848272	KC848357	-----	-----	-----
*Trametes palisotii*	OAB0118	Benin	MK736980	MK736956	MK802884	MK802882	MK802886
*Trametes palisotii*	OAD0153	Benin	MK736981	MK736957	MK802885	MK802883	MK802887
*Trametes palisotii*	OAD0198	Benin	MK736982	MK736958	-----	-----	MK802888
*Trametes palisotii*	DMC360	Cameroon	KC589139	KC589160	-----	-----	-----
*Trametes palisotii*	DMC817	Cameroon	KC589142	KC589163	-----	-----	-----
*Trametes palisotii*	DMC816	Cameroon	KC589141	KC589162	-----	-----	-----
*Trametes parvispora*	OAB002	Benin	MK736989	MK736964	-----	MN127965	-----
*Trametes parvispora*	OAB0023	Benin	MK736990	MK736965	-----	MN127964	-----
*Trametes polyzona*	DMC370	Cameroon	KC589125	KC589151	-----	-----	-----
*Trametes polyzona*	Cui 11040	China	KX880647	KX880689	KX880836	KR610849	-----
*Trametes polyzona*	BKW004	Ghana	JN164978	JN164790	-----	-----	-----
*Trametes polyzona*	OAB0092	Benin	MK736984	MK736959	-----	-----	-----
*Trametes polyzona*	OAB0128	Benin	MK736985	MK736960	-----	-----	-----
*Trametes polyzona*	OAB0195	Benin	MK736986	MK736961	-----	-----	-----
*Trametes pubescens*	FP101414sp	USA	JN164963	JN164811	JN164827	JN164851	-----
*Trametes punicea*	BCC26408	Thailand	JF372685	FJ372707	-----	-----	-----
*Trametes punicea*	BCC27595		FJ372686	FJ372708	-----	-----	-----
*Trametes rependa*	FRI437T		JN164985	-----	KF573177	KF573142	KF573080
*Trametes rependa*	FPRI390	Philippines	JN164921	-----	KF573175	KF573141	KF573077
*Trametes rependa*	OH271sp	Venezuela	JN164936	-----	KF573176	KF573143	KF573079
*Trametes rependa*	M0138339	Papua New	KF573029	-----	-----	KF573140	KF573078
*Trametes sanguinea*	OAB0088	Benin	MK736969	MK736949	-----	-----	-----
*Trametes sanguínea*	PRSC95	Puerto Rico	JN164982	JN164795	JN164842	JN164858	-----
*Trametes sanguínea*	BCC 36861	Thailand	GQ982885	GQ982878	-----	-----	-----
*Trametes sanguínea*	8R12	Thailand	FJ372672	FJ372694	-----	-----	-----
*Trametes sanguinea*	CBS61473	Sri Lanka	MH860781	MH872513	-----	-----	-----
*Trametes socotrana*	BJFC12724	China	KC848313	KC848397	-----	-----	-----
*Trametes* sp.	BC1	Finland	KT896651	-----	-----	-----	-----
*Trametes suaveolens*	FP102529sp	USA	JN164966	JN164807	JN164828	JN164853	-----
*Trametes suaveolens*	Dai 10729	China	JN048770	JN048789	-----	-----	-----
*Trametes versicolor*	FP13515sp	USA	JN164919	JN164809	JN164825	JN164850	-----
*Trametes villosa*	FP71974R	USA	JN164969	JN164810	JN164830	JN164855	-----

The primers used for ITS were ITS1 and ITS2 and for LSU were LROR-LR3 and LRr [17]. Additionally, three protein-coding genes for RNA polymerase II were included, they were *rpb1*, the largest subunit and *rpb2* the second largest subunit (RPB2-5F/RPB2-7cR) and translation elongation factor 1-α (*tef1*, 983F- 2218R; [18]).

## Data Availability

The data that support the findings of this work are available from the corresponding author upon reasonable request. The sequenced amplicons are available on NCBI. The data underlying this article will be shared at reasonable request to the corresponding author.
